# Case Report: Primary catastrophic antiphospholipid syndrome in a pediatric patient with cerebral venous sinus thrombosis as the first manifestation

**DOI:** 10.3389/fped.2024.1491095

**Published:** 2024-12-11

**Authors:** Lingyang Xu, Jing Wu, Haidong Wang, Baowang Yang

**Affiliations:** Pediatric Intensive Care Unit, The Second Hospital of Lanzhou University, Lanzhou, China

**Keywords:** case report, primary catastrophic antiphospholipid antibody syndrome, multiple thrombus, children, cerebral venous sinus thrombosis

## Abstract

**Background:**

Antiphospholipid syndrome (APS) is an autoimmune disease characterized by recurrent vascular thrombotic events. Catastrophic APS (CAPS), which can result in multiple organ failure and even death, is the most severe manifestation of APS. Herein, we report the case of a pediatric patient with CAPS, including the clinical course, diagnosis, and treatment, with the goal of expanding the literature on this condition, as reports of CAPS in pediatric patients are rare.

**Case presentation:**

A 7-year-old girl presented with cranial hypertension. She was initially admitted to the hospital with a diagnosis of cerebral venous sinus thrombosis (CVST) and was discharged following symptom improvement. However, only 3 days later, the patient was re-presented with cranial hypertension and multiple thromboses and was ultimately diagnosed with CAPS based on multidisciplinary consensus. Despite treatment with a series of anticoagulation and thrombolytic therapies, the child's condition progressed rapidly, and she eventually died of pulmonary embolism.

**Conclusion:**

CAPS in children is rare and associated with a high mortality rate, making early recognition and diagnosis critical but difficult. Based on the presented case, we recommend routine screening for antiphospholipid antibodies in children with CVST without obvious triggers, or a multidisciplinary collaboration, to facilitate the early diagnosis of CAPS.

## 1 Introduction

Antiphospholipid syndrome (APS) is a non-inflammatory autoimmune disease characterized by recurrent vascular thrombotic events and spontaneous abortions and/or stillbirths, in addition to thrombocytopenia, with moderate or high positivity for antiphospholipid antibodies (1). This condition can be subcategorized as primary or secondary APS. The 2006 Sydney diagnostic criteria for APS, which are well-recognized internationally, include clinical criteria (arterial or venous thrombosis of any organ or tissue and pathological pregnancy) in addition to laboratory criteria (positive lupus anticoagulant, anti-cardiolipin antibody, or anti-beta2-glycoprotein 1 antibody), with meeting one of each of the clinical and laboratory criteria satisfying the requirements for diagnosis of APS. The incidence of APS is approximately 5 new cases per 100,000 persons per year, and the prevalence is approximately 40–50 cases per 100,000 persons (2). In 2023, the American College of Rheumatology (ACR) and the European Society of Rheumatology (EULAR) jointly developed a novel classification system for APS that includes the inclusion criteria from both organizations, with additional weighting criteria (3). The most severe manifestation of APS is catastrophic APS (CAPS), which is characterized by rapid progressive and extensive thrombosis, potentially resulting in multi-organ failure and even death.

Cerebral venous sinus thrombosis (CVST) is a unique cerebrovascular condition characterized by intracranial hypertension due to obstruction of the cerebral venous reflux, commonly accompanied by impaired absorption of the cerebrospinal fluid (CSF), due to a variety of etiological factors. The incidence of CVST in children is 7 per 1 million individuals (4). Infection, including sinusitis, otitis media, mastoiditis, purulent meningitis, and sepsis, is the most common cause of CVST in children. Non-infectious factors are primarily related to cranial trauma, dehydration, arteriovenous malformations, hematological disorders, immune system disorders, and coagulation system abnormalities.

Existing case reports of CAPS have predominantly described cases in adults. CAPS in children is extremely rare, and it is easily missed or misdiagnosed in clinical practice. Although cases in children are rare, the fatality rate is very high (33%–50%) (5). Herein, we report a rare case of primary CAPS in a child who presented with CVST and within a short time developed intracranial CVST, deep vein thrombosis in both lower extremities, and pulmonary embolism due to rapid disease progression, consistent with the diagnosis of CAPS. We report this case with the goals of deepening the understanding of this disease among pediatricians, neurosurgeons, and rheumatologists as well as furthering the study of CAPS in children.

## 2 Case description

**Figure 1:**
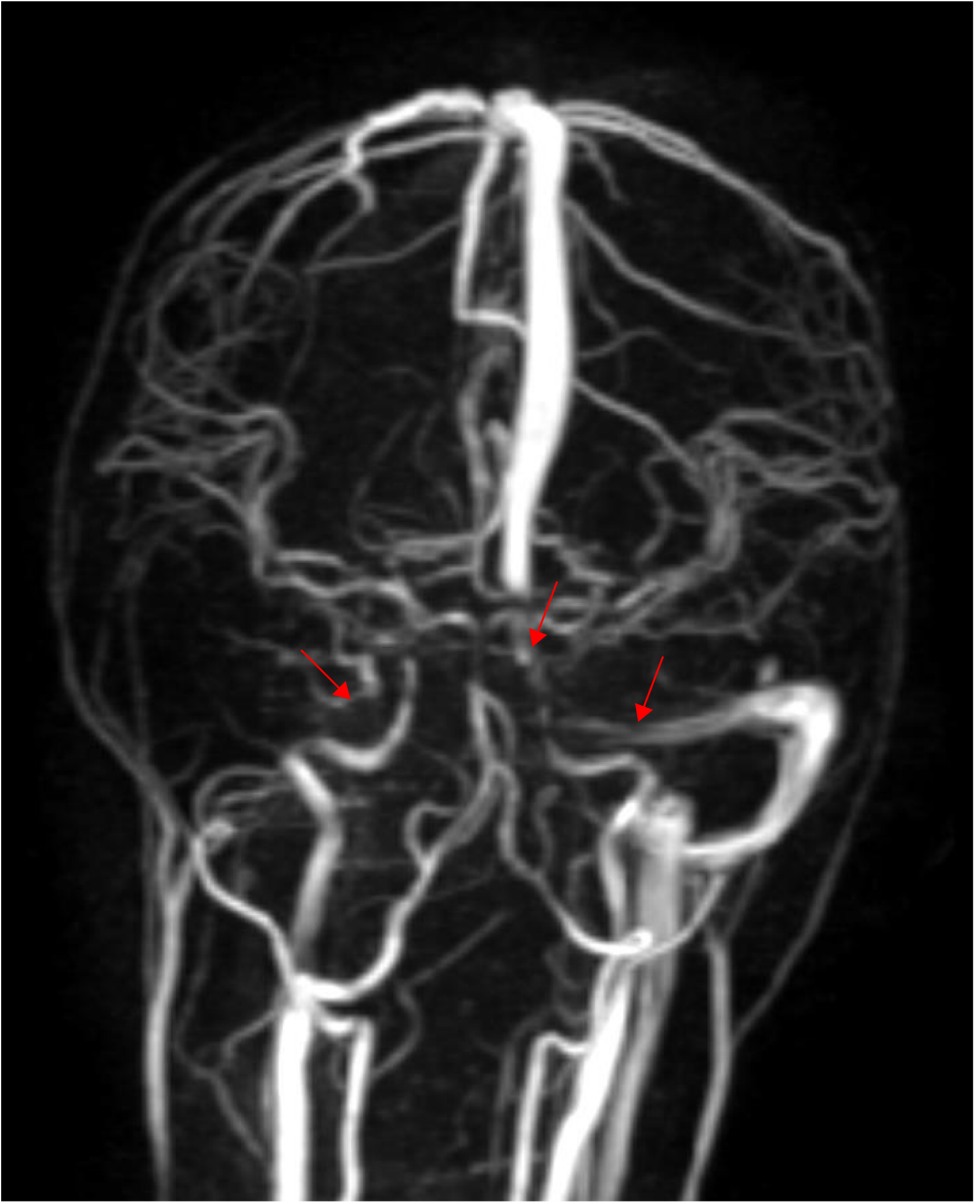
Thrombosis of sinus confluence, right transverse sinus and sigmoid sinus, and left transverse sinus (performed on June 6, 2024).

A 7-year-old girl was admitted to the Second Hospital of Lanzhou University in June 2024 after experiencing headache with nausea, vomiting, and neck pain for 4 days. These symptoms had started without any obvious triggers. During this period, she experienced projectile vomiting of the gastric contents 3–4 times/day, with no fever, convulsions, or consciousness disorders. The patient was admitted to a local county hospital where she was found to have a blood-like, hyperdense shadow in the right transverse and sigmoid sinuses on computed tomography (CT) examination. The patient was admitted to our hospital for further diagnosis and treatment. The patient had normal growth and development, with no notable medical history. Physical examination at admission revealed that the patient had stable vital signs, clear consciousness, poor spirit, headache, stiff neck pain, and bilateral positive Pap sign along with a prothrombin time (PT) of 12.9 s (reference range: 9.4–12.5 s), International Normalized Ratio (INR) of 1.20 (reference range: 0.85–1.14), D-dimer concentration of 7.47 μg/ml (reference range: <0.50 μg/ml), and fibrin degradation products (FDP) concentration of 22.48 μg/ml (reference range: <5.00 μg/ml). The patient was positive for antinuclear antibodies. No abnormalities were found on cerebrospinal fluid pressure measurement, biochemical tests, routine blood tests, or culture testing. Cranial magnetic resonance imaging (MRI) revealed that the signals in the sinus cavity of the sinus confluence, right transverse sinus, and ethmoidal sinus were not homogeneous. Magnetic resonance venography (MRV) showed sinus confluence, right transverse sinus and sigmoid sinus thrombosis, and left transverse sinus thrombosis (Figure 1).

**Figure 2:**
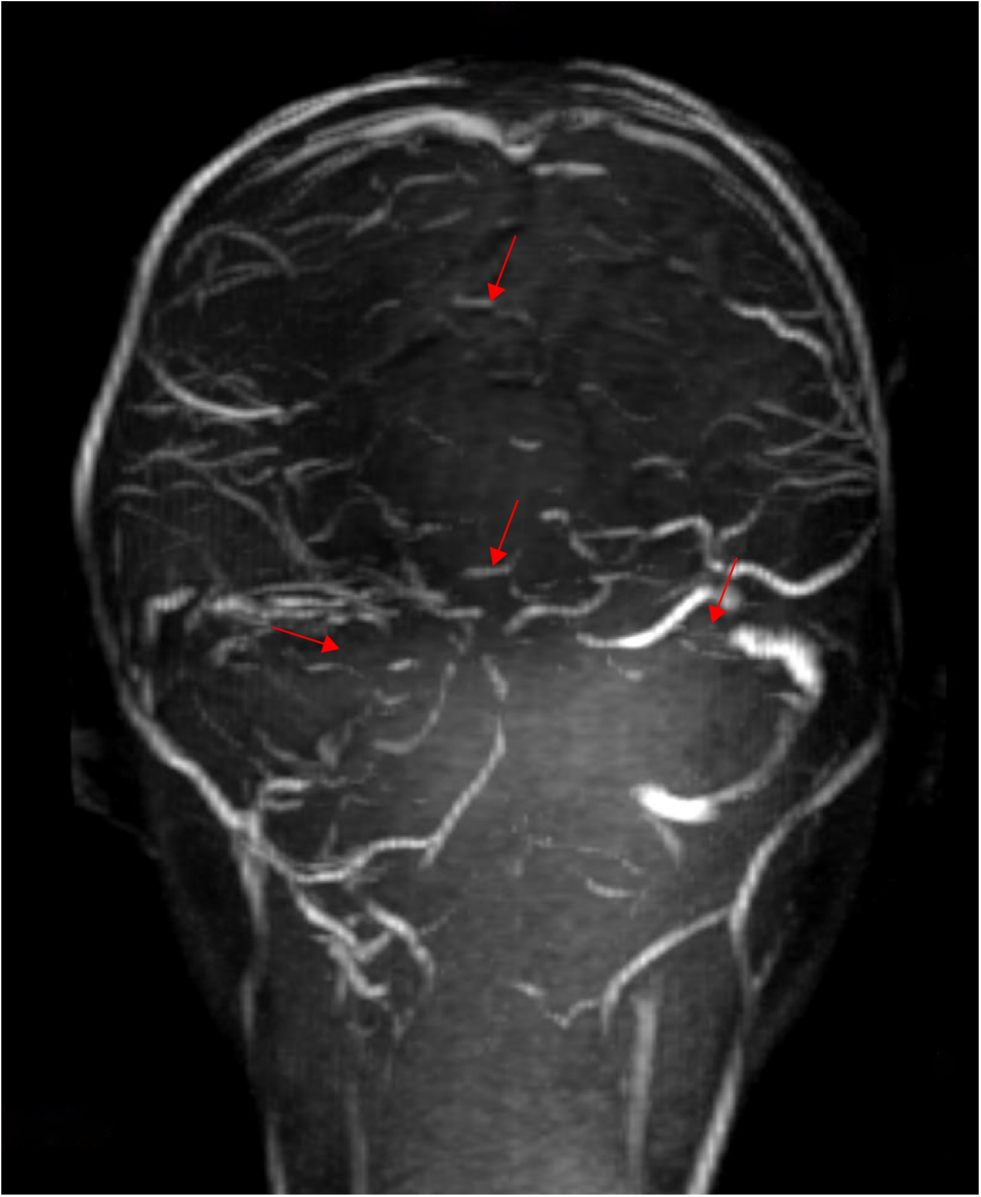
Thrombosis in the superior sagittal sinus, straight sinus, right transverse and sigmoid sinus, right internal jugular vein, left transverse sinus, and sinus confluence, with an increased extent of thrombosis compared with the previous MRV (performed on June 6, 2024).

**Figure 3:**
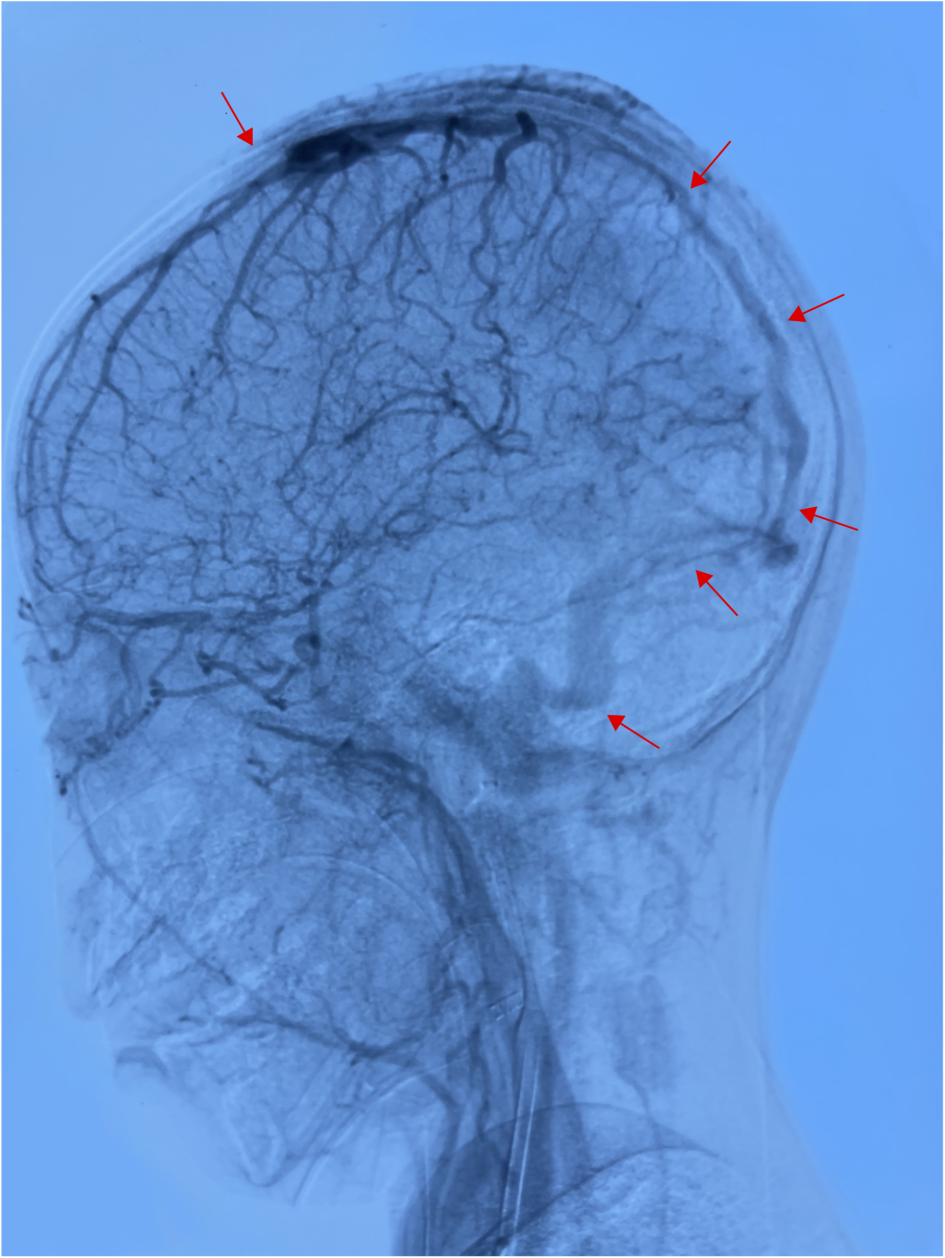
After thrombectomy, the superior sagittal sinus was not developed in the whole process, and the bilateral transverse sinus and sigmoid sinus were unobstructed (performed on June 23, 2024).

**Figure 4:**
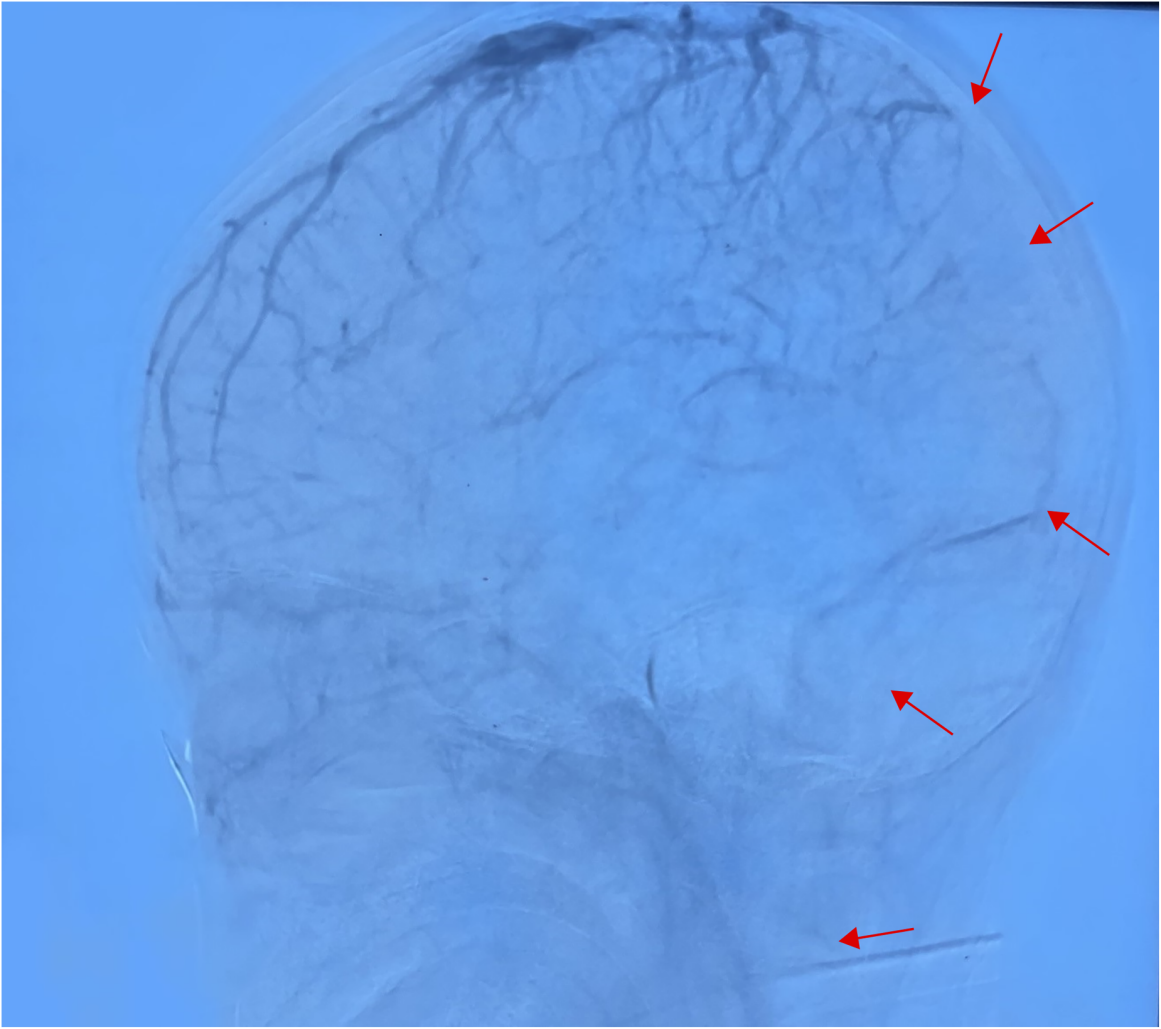
In the venous sinus stage, the superior sagittal sinus was not developed throughout, and the bilateral transverse and sigmoid sinuses were poorly developed (performed June 28, 2024).

Based on the above presentations, the patient was diagnosed with non-purulent thrombosis of the intracranial venous sinus. The first time the child was admitted to the hospital, we gave: mannitol 100 ml, q8h*13 days; enoxaparin sodium 2500 AxaIU, ih, q12h*13 days; ceftizoxime sodium 1.0 g, q8h*7 days; and rivaroxaban 10 mg/dose, qd*3 days. The child was hospitalized for 14 days and was discharged after her neurological symptoms subsided, with a recommendation to take rivastigmine 10 mg qd orally. However, 3 days after discharge, the child was admitted to the hospital again with recurrence of headache and vomiting for the previous 6 h. MR cranial enhancement revealed thrombosis of the superior sagittal sinus, straight sinus, right transverse sinus, ethmoidal sinus, right internal jugular vein, and sinusoidal confluence, while the scope of the thrombus had increased compared with that on the previous MRV examination (Figure 2). Following neurosurgery consultation, cerebral angiography was performed (Figure 3), combined with transcatheter intracranial vascular thrombectomy, after which the internal jugular vein and intracranial venous sinus were placed in a microcatheter, and pumped with urokinase to dissolve the thrombus. Subsequently, the patient was provided with respiratory support, sedation, analgesia, anti-infection therapy, dehydration and lowering of the cranial pressure, and enoxaparin anticoagulation therapy, among other treatments. Further, on the 4th day postoperatively, cranial CT was repeated, with the results indicating that the range of the thrombus in the intracranial venous sinus had increased slightly compared with that on the previous scan. Cerebral angiography was repeated on the first postoperative day after the operation (Figure 4). Postoperatively, the child's bilateral pupils were unequal in size, with the left pupil measuring 4 mm in diameter and the right pupil measuring 2 mm in diameter and both with a dull light reflex. Therefore, cranial hypertension was considered, and mannitol and albumin were administered to lower the cranial pressure via dehydration and tachypnea. The child's bilateral pupil sizes and reflexes returned to normal, and the respirator was withdrawn.

Further examinations revealed the following results. On lupus anticoagulant assay (LA), the LA diluted Russell viper venom time (dRVVT) was 54.10 s, with an LA dRVVT confirmation of 33.30 s and dRVVT ratio of 1.44 (reference range: <1.2). Antinuclear antibody (ANA) testing was positive, with a cytoplasmic value of 1:80 (reference range: negative). Tests for thrombophilia showed: thrombin-antithrombin complex, 43.8 ng/ml (reference range: <4 ng/ml); fibrin-α2 fibrinolysis inhibitor complex, 10.5 μg/ml (reference range: <0.8 μg/ml), and thrombomodulin and tissue plasminogen activator-inhibitor 1 complex levels in the normal ranges. Testing for anti-phosphatidylethanolamine antibody IgM was positive. The levels of anti-cardiolipin antibodies against IgA, IgG, IgM, and β2 glycoprotein 1 (IgGAM) were all within the normal ranges (Table 1). Protein C and S levels were also within the normal ranges. Vascular ultrasonography revealed thrombosis of the right internal jugular vein and bilateral thromboses of the femoral and external iliac veins. Based on the results of the examination, a final diagnosis of APS with intracranial venous sinus thrombosis and bilateral lower extremity deep vein thrombosis was made. The patient was subsequently treated with prophylaxis, hormones, low molecular weight heparin calcium and aspirin as anticoagulation therapy, plasma, dehydration therapy, and lowering of the cranial pressure. Following this treatment, the child was conscious with stable vital signs, and the family requested discharge for the patient to receive further treatment at a more specialized hospital if her condition allowed. The child was discharged to a hospital in Beijing, where she died of a pulmonary embolism after 4 days of hospitalization.

**Table 1:** Laboratory findings after re-admission to the hospital.

Test item	Result	Normal range
PT	12.6	9.4–12.5 s
INR (on admission)	1.17	0.85–1.14
INR (5 days later)	1.44	
INR (at discharge)	1.06	
D-Dimer (on admission)	7.97	<0.50 µg/ml
D-Dimer (5 days later)	129.07	
D-Dimer (at discharge)	3.69	
FDP (on admission)	21.72	<5.00 µg/ml
FDP (5 days later)	269.63	
FDP (at discharge)	5.67	
PLT count	291	167–453*10^9 ^/L
LA (S/C)	1.44	<1.20
ANA	Positive	Negative
Cytoplasmic	1:100	Negative
dsDNA	Negative	Negative
ACA-IgA	0.47 APLU/ml	10 APLU/ml
ACA-IgG	1.53 GPLU/ml	<10 GPLU/ml
ACA-IgM	2.56 MPLU/ml	<10 MPLU/ml
anti-β 2-glycoprotein 1	<20.00 RU/ml	<20 RU/ml
AT-III	106%	83%–128%
Protein C	88.0%	70.0%–140.0%
Protein S	111.1%	63%–150%
Homocysteine	1.7 µmol/L	4–15 µmol/L
TAT	43.8 ng/ml	<4 ng/ml
PIC	10.5 µg/ml	<0.8 µg/ml
TM	8.10 TU/ml	3.8–13.3 TU/ml
t-PAIC	2.60 ng/ml	<10.50 ng/ml

Abbreviations: PT, prothrombin time; INR, International normalized ratio; FDP, fibrinogen degradation products; PLT, platelet; LA (S/C), lupus anticoagulant; ANA, antinuclear antibody; dsDNA, double-stranded DNA; ACA-IgA, anti-cardiolipin antibody-IgA; ACA-IgG, anti-cardiolipin antibody-IgG; ACA-IgM, anti-cardiolipin antibody-IgM; AT-III, antithrombin III; TAT, thrombin antithrombin complex; PIC, plasmin-α2 plasmin inhibitor complex; TM, thrombomodulin; t-PAIC, tissue plasminogen activator-inhibition 1 complex.

## 3 Discussion

Thrombotic events are a common clinical manifestation of APS and can occur in almost any vessel, with deep vein thrombosis in the lower extremities being the most common, while intracranial venous sinus thrombosis is relatively rare (5). One prior retrospective study reported that the prevalence of CVST in patients with APS was 0.7% (6). Owing to the diverse and nonspecific clinical manifestations of APS, early diagnosis remains a significant challenge in clinical practice.

CAPS in children has an insidious onset and varying clinical manifestations, making differential diagnosis critical and complex. Neuro-Behcet's disease (NBD) is a neurological disorder in patients with Behcet's disease (BD). The clinical manifestations are diverse and lack specificity. The most common manifestations of NBD can be divided into a parenchymal type and CVST. A study of NBD in children found that the most common type of nervous system involvement was CVST, which was confirmed by MRI and MRV in 23 cases (88.5%) (7). Regarding systemic manifestations of BD, oral ulcers, skin lesions, and genital ulcers are the most common symptoms in pediatric NBD patients. In the presented case, CVST was the first manifestation, and thus, differentiation from NBD was necessary. Because oral ulcers, genital ulcers, and skin lesions were not found on physical examination, NBD was not considered for diagnosis.

CAPS can also be easily misdiagnosed as systemic lupus erythematosus (SLE) when the onset is renal and multi-system damage is detected. Canpolat et al. reported a case of CAPS in a child who was misdiagnosed as SLE (8). The adolescent girl (14 years old) presented with suspicion of SLE and was admitted to the hospital with negative ANA and normal anti-dsdna. Renal biopsy revealed no immune deposits. A diagnosis of SLE was ruled out. Parvovirus B19 IgM and IgG were positive. The anticardiolipin (aCL) antibody level was elevated. Because there was no evidence of thrombosis, high levels of aCLs were considered to be associated with persistent infection. She was diagnosed with vasculitis associated with parvovirus B19 infection, which primarily affects the kidneys, heart, and eyes. Upon treatment with human immunoglobulin and prednisone, her condition improved, and she was discharged from the hospital. Nine months later, she was readmitted due to weakness, headache, and palpitations. At that time, she had high anticardiolipin antibodies, heart failure, retinal microthrombosis, and small vessel occlusion confirmed by renal biopsy histopathology. Based on these clinical and laboratory findings, she was diagnosed with CAPS. Antiphospholipid antibodies can arise transiently during viral infections, and treatment with corticosteroids and intravenous immunoglobulin (IVIG) masked the condition, which contributed to the misdiagnosis on her initial admission.

Indeed, APS can be divided into primary APS and secondary APS. SLE is the most common type of secondary APS. Juvenile SLE (jSLE) is considered a rare disease, with an incidence of 0.3–0.9 cases per 100,000 children and a prevalence of 1.89–25.7 cases per 100,000 children. A study of 92 children with jSLE showed that the percentages of patients positive for ANA and anti-dsdna were 97.8% (*n* = 90) and 84.8% (*n* = 78), respectively. aCL IgM and/or IgG antibodies were present in 12% (*n* = 11) of the cases in which testing for antiphospholipid antibodies was conducted (9). Only 3 cases (3.3%) were classified as APS. Therefore, in clinical practice, we should also routinely screen for antiphospholipid antibodies in patients with jSLE to determine whether APS is secondary.

At the onset of this pediatric case, the patient presented with the classic cranial hypertension symptoms of headache and vomiting, which is consistent with the results of a study of 21 cases of APS combined with CVST (10), in which headache (90.5%) was the most common neurological symptom. The child underwent head CT scanning at an external hospital, which revealed a blood-like hyperdense image in the right transverse and sigmoid sinuses. Following her transfer to the pediatric intensive care unit of our hospital, cranial MRI and MRV revealed intracranial venous sinus thrombosis. A previous study (7) reported that the most frequently involved sites of CVST are the transverse sinus (76.2%) and superior malleolar sinus (57.1%), followed by the ethmoid sinus (52.4%), the internal jugular vein (38.1%), and the straight sinus (14.3%).

Because the most common cause of CVST in children is infection, the patient was first admitted to the hospital to receive low molecular weight heparin anticoagulation therapy, cranial pressure reduction therapy, and anti-infection treatment. Due to the lack of sufficient knowledge of the etiology of CVST, no other immunological investigations were performed, and the child was therefore subsequently discharged with oral anticoagulant treatment. Three days post-discharge, the child was re-admitted to the hospital with recurring symptoms of cranial hypertension, and the intracranial venous sinus thrombus was found to have increased significantly. Then it was determined that the cause of the child's CVST was not an infection, but rather something else. We then conducted thrombophilia testing, antiphospholipid antibody testing, lower extremity vascular ultrasound, and other related examinations and invited physicians in the rheumatology, vascular surgery, neurosurgery, and interventional medicine departments to conduct multidisciplinary discussions, which resulted in a definitive diagnosis of CAPS.

Upon consultation with neurosurgery after the diagnosis of CVST, angiography and intracranial thrombectomy were performed, with the resulting supporting the diagnosis of CAPS. We then reviewed the literature and found that surgery is not beneficial to the prognosis of children with CAPS. The treatment of CAPS in children includes anticoagulation therapy, glucocorticoids, and cytotoxic drugs such as cyclophosphamide, combined with plasma exchange or intravenous gamma globulin (11). We administered anticoagulation therapy, glucocorticoids, and intravenous gamma globulin in the treatment of this patient with CAPS. Before plasma exchange could be started, the patient's guardians requested her transfer to another hospital.

From our literature review, we concluded that the clinical manifestations of APS with CVST can vary. In one study of 624 patients with CVST (12), the potential etiology of CVST in patients with systemic inflammatory diseases included APS, inflammatory bowel disease, leukemia, and SLE in 5.9%, 1.6%, 1%, and 1% of the cases, respectively. Conversely, in a series of 1,000 patients with APS published by Cervera et al. (6), CVST was present in only 7 patients. Thus, CVST is an uncommon manifestation in patients with APS, and most of the published studies are either case reports or short case series, with reports in children being even rarer. In one study of 27 patients with APS combined with CVST (13), only two patients developed both deep vein thrombosis and pulmonary thromboembolism. Because APS combined with CVST is rare in pediatric patients, lacking a specific clinical presentation, missed and misdiagnoses are common, and a multidisciplinary collaboration may be required for early diagnosis.

Our review of the literature also revealed that anticoagulant therapy and corticosteroids are the two main treatments for CAPS in children. In an analysis of 45 CAPS case reports in children (14), anticoagulant therapy (ACs) was the most commonly used treatment (35/45 cases, 77.8%). Corticosteroids (CS) were used in 34 of the 45 cases (75.6%), as well as plasma exchange (PE) in 18 cases (40.0%), IVIG in 17 cases (37.8%), and cyclophosphamide in 11 cases (24.4%). Most of the patients received comprehensive treatment. ACs + CS was the most common combination (20.9%), followed by ACs + CS + PE and/or IVIG (18.6%), and ACs + CS + cyclophosphamide + PE and/or IVIG (11.6%). In terms of mortality, 12 patients (26.1%) died at the time of the catastrophic event. Of note, all patients treated with the combination of ACs + CS + PE and/or IVIG survived the catastrophic event; however, the survival difference was not statistically significant.

In general, anticoagulants are the first-line drugs for the treatment of APS (15), with most clinicians prescribing warfarin to maintain the INR at 2–3. Aggressive anticoagulant therapy for CAPS can be combined with high-dose hormones, gamma globulin, cyclophosphamide, and plasma exchange to improve patient survival. Surgical thrombectomy may further exacerbate the embolization of CAPS caused by exogenous infections (16). Successful hematopoietic stem cell transplantation has also previously been reported (17). However, there are currently no clear guidelines for the treatment of CAPS in children; therefore, in treating the patient described herein, we referred to the protocol for adults and administered intravenous human immunoglobulin, hormones, anticoagulation therapy, and other treatments, followed by venous thrombectomy. The apparent thrombus at the site of tube placement after the operation was attributed to disease progression; however, the possibility this was due to surgical stimuli could not be ruled out. Although was learned that the patient in this case died of pulmonary embolism 4 days after admission to another hospital, we were not able to obtain any laboratory test results, examination results, and treatment plan details from that hospital, which represents another limitation and insufficiency of this study. Further clinical studies are required to evaluate the treatment of pediatric CAPS.

A previous study (18) found that after screening for antiphospholipid antibodies in three pediatric patients with CVST as the clinical manifestation, all three patients were finally diagnosed with APS. Therefore, this study suggests that pediatric patients with CVST should be screened for antiphospholipid antibodies and lupus anticoagulation in order to reduce missed diagnosis and misdiagnosis (19).

## 4 Conclusions

Reports of CAPS in pediatric patients with the development of CVST simultaneously complicated by thrombosis in three or more organs are rare in both the Chinese and international literature. In the present case, the patient presented with cranial hypertension caused by CVST and subsequently developed thrombosis of the internal jugular vein and deep veins of both lower extremities within a short period. Based on the presented case and the corresponding literature review, we recommend routine screening for antiphospholipid antibodies in children with CVST without obvious triggers, or a multidisciplinary collaboration, to facilitate the early diagnosis of CAPS, which can help improve the survival rate among pediatric patients.

## Data Availability

The original contributions presented in the study are included in the article/Supplementary Material, further inquiries can be directed to the corresponding author.
